# Scoping Review of Technological Solutions for Community Dwelling Older Adults and Implications for Instrumental Activities of Daily Living

**DOI:** 10.14336/AD.2024.0215

**Published:** 2024-02-15

**Authors:** Umut Arioz, Urška Smrke, Nejc Plohl, Tanja Špes, Bojan Musil, Izidor Mlakar

**Affiliations:** ^1^The University of Maribor, Faculty of Electrical Engineering and Computer Science, Maribor, Slovenia.; ^2^The University of Maribor, Faculty of Arts, Department of Psychology, Maribor, Slovenia.

**Keywords:** aging adults, aging in place, technological solutions, scoping review, instrumental activities of daily living

## Abstract

Aging in place is not without its challenges, with physical, psychological, social, and economic burdens on caregivers and seniors. To address these challenges and promote active aging, technological advancements offer a range of digital tools, applications, and devices, enabling community dwelling older adults to live independently and safely. Despite these opportunities, the acceptance of technology among the older adults remains low, often due to a mismatch between technology development and the actual needs and goals of seniors. The aim of this review is to identify recent technological solutions that monitor the health and well-being of aging adults, particularly within the context of instrumental activities of daily living (IADL). A scoping review identified 52 studies that meet specific inclusion criteria. The outcomes were classified based on social connectedness, autonomy, mental health, physical health, and safety. Our review revealed that a predominant majority (82%) of the studies were observational in design and primarily focused on health-related IADLs (59%) and communication-related IADLs (31%). Additionally, the study highlighted the crucial role of involving older adults in study design processes, with only 8 out of the 52 studies incorporating this approach. Our review also established the interview method as the most favoured technology evaluation tool for older adults' studies. The metrics of 'usability' and 'acceptance' emerged as the most frequently employed measures for technology assessment. This study contributes to the existing literature by shedding light on the implications of technological solutions for community dwelling older adults, emphasizing the types of technologies employed and their evaluation results.

## 1. INTRODUCTION

The proportion of aging adults is rapidly increasing globally. By 2050, it is expected that 16% of the world's population will be over 65 years old [1]. This shift is a significant social transformation with far-reaching implications, particularly for healthcare systems [2].

Current healthcare systems attempt to address the evolving needs of the older adults by offering institutionalized living environments with various services. However, transitioning from a home environment to an institutionalized one can be a challenging and potentially traumatic experience for the older adults [3]. Research by Kerbler et al. highlights the positive effects of remaining in a familiar environment on mental and physical health, as well as overall quality of life. Living in one's own home supports self-esteem, identity, independence, security, intimacy, belonging, and community relationships [4].

Despite the goal of aging in place with these positive effects dating back to the 1980s [5], older adults individuals who choose to remain in their home environment may encounter various challenges, affecting not only them but also their caregivers, relatives, and friends. Informal care is required by most of the older adult’s population, approximately 70%, imposing significant physical, psychological, social, and economic burdens [6]. To ensure well-being and quality of life across all life stages, existing technological systems must adapt to support the changing demographic structure and promote opportunities for active aging [7].

Seniors often encounter physical and psychological challenges as they grow older in their accustomed surroundings. These challenges can manifest as difficulties with mobility, declining cognitive function, and heightened vulnerability to health problems, thereby affecting their capacity to live autonomously. Advances in technology offer a range of digital tools, applications, and devices (e.g., Internet of Things, artificial intelligence, assistive robotics) that enable community dwelling older adults to live independently and safely in their accustomed surroundings [6, 8, 9]. However, the acceptance rates of these technologies among the older adults remain low. A key reason for this issue is the failure to consider the actual needs and goals of the aging population in the development of Ambient or Active Assisted Living (AAL) solutions [8].

### Technologies to Support community dwelling older adults for healthy aging

A great interest and willingness to contribute to the efficiency of in-home care via different technologies are still ongoing in medical informatics. Due to several challenges, technology implementations in the real-life could not reach to expected level. Not having an error-free and high reliable prototype, needing an interdisciplinary work and having many requirements to adopt the technologies in home environment are the main challenges of health-enabling technologies at home [10].

In the literature, few review studies analysed aspects of health-enabling technologies, like telemonitoring for older adults [11], home-monitoring systems for patients with heart-failure [12], tele-medicine applications for geriatrics department [13] and benefits of home telecare applications [14, 15]. The first comprehensive review study on health-enabling studies for older adults was published by [10], by providing an overview of services for those technologies and their archetypical categories. Based on their findings, there are two broad range of technologies for older adults: (1) technologies related with handling adverse conditions which includes emergency calls, automated detection of deviant behaviour, falls and cardiac emergencies, and handling dangerous situations; and (2) technologies related with assessing the state of health which includes early recognition of some diseases and medical conditions and monitoring of existing diseases and therapeutic interventions.

In recent years, the classification studies of in-home technologies from technical perspective were conducted [16, 17]. Following categories were recognised: *monitoring daily activities, monitoring abnormal behaviours, monitoring cognitive impairment, fall detection, indoor person localization and monitoring sleep quality*. Under these categories many non-invasive and non-intrusive devices and different technologies were offered to support community dwelling older adults. Between these devices, television was used to provide comfortable set up at home and to increase the user acceptance, especially for older adults [18]. Wearable devices which are one of the most common and rapidly growing technology may help in changing behaviour and to increase the quality of life of older adults [19]. They are mainly used to monitor position in home, to recognize the activities of older adults and to monitor vital measurements in real-time [20]. Another growing interest is to use robot assistants especially where there are not any caregivers to help older adults. Although those robots have still some challenges to be solved technically and socially, they have potential to provide autonomy and self-sufficiency feelings to older adults [21, 22]. Embodied conversational agent is used in specific areas mainly for improving the quality of life as ease of living for dementia patients [23]. Sensors which are embedded or attached to any part of the home are the main components of in-home monitoring, and they can provide not only support to older adults about their daily activities but also decrease burden on caregivers by remote monitoring [24]. Kim et al. specified 16 different types of sensors with data they provide and usage locations at home, and the first three most common sensors are motion sensors (at walls or ceiling for specific activities and walking), contact sensors (at doors, windows, refrigerators, etc. for security and usage) and pressure sensors (at chairs, floor, beds, etc. for measuring the duration of activities such as sleeping or sitting) [16].

Smart home monitoring technologies can be counted as an integrated solution for older adults. With the increase of IoT (internet of things) technology with more reliable devices and sensors, the usage areas of smart home monitoring technologies for health have also increased in recent years [25] such as: physiological monitoring for vital signs such as blood pressure, sugar level and body temperature [26, 27]; functional monitoring for daily activities such as walking and food intake [28]; emergency detection for fallings [29]; safety monitoring such as preventing gas leak or fire [30]; security monitoring such as possible human threats or detection of suspicious cases [31]; socializing monitoring such as interaction via phone or online video [32]; and cognitive assistance such as medication reminder, guidance for lost items [33]. According to the study of [25], most of the smart home monitoring technologies was focused on functional monitoring (activities of daily living (27.1%), health-related quality of life (22.9%) or falls (16.7%)).

The effect of mobile technology on health interventions was reviewed by a few studies in the literature [34, 35]. Although Louras et al. reviewed the heterogeneity of the study designs and evaluation metrics [35], they showed the positive contribution of mobile technology integration into health interventions by reduced emergency calls, emergency transportation rates and healthcare costs with increased patient experience. The usage of mobile technologies for cognitive assessment of older adults was reviewed by Koo et al. [34] and showed that mobile technology has a potential to help assessment and collection of behavioural markers which are important for cognitive impairment. On the other hand, the problems of usage o smart phones and tablets in health interventions were also summarized in the literature. For example, potential solutions for easy adaptation and high user engagement of mobile technologies into health interventions were reviewed by van Acker et al. [36]. In this review study, it was emphasized that limitation factors such as physical, psychological, and motivational barriers should be considered for the future implementations. Wilson et al. listed the problems of technology usage by older adults and specified the need for further studies to develop evidence-based techniques for cognitive impairment [37].

In the recent years, in line with WHO definition [38], the assistive technologies for older adults gained new classification perspective besides technical approach - potential outcomes of the technologies which was expressed by Rolfe et al. [39]. In this study, each technology was classified according to having different outcomes like *social connectedness, autonomy and independence, mental health and wellbeing, physical health and safety.* Although some technologies may have impact on more than one outcome, this categorization can be used as a starting point for older adults to examine technology options. Social connectedness is based on communication with family and friends by using text, video or video via laptops, mobile phones, or PCs. In the autonomy and independence category, the main is to control the daily life and living environment by reminding and assistance technologies via mobile applications or smart home solutions [40, 41]. Mental health and wellbeing category includes technologies improving cognitive capacity and providing emotional and mental health [42]. Brain games, online training programmes and some online information sources can be counted within this category. In physical health category, technologies related to physical exercise, management of health conditions and medication tracking are considered such as exergames [43], online exercise programmes, health consultation applications, monitoring applications for blood pressure, step counting, etc. Safety category includes technologies for monitoring risks to physical health and social or financial situations. Alert systems, smart home devices, fall detection systems, sensors networks [44] and security cameras (e.g. for dementia patients [45] fall into this category.

### Studies on BADL and IADL

Older adults’ overall competence or functional capacity significantly impact their quality of life during the aging process, consequently, determine their ability to make independent decisions and carry out everyday activities [46]. The level of these capabilities relates with the degree of assistance required to perform daily tasks. In the literature, there are two distinct categories for activities of daily living (ADL): basic activities of daily living (BADL) and instrumental activities of daily living (IADL).

BADLs encompass essential activities necessary for independent living at home, such as bathing, dressing, eating, and feeding. In contrast, IADLs involve more complex tasks like shopping, making telephone calls, preparing meals, and managing finances. IADLs demand a higher level of autonomy and the performance of executive functions, including attention, working memory, problem-solving, planning, and mental flexibility [47].

The performance level in both BADLs and IADLs serves as an indicator of disability in aging adults [48]. Furthermore, the performance in BADLs and IADLs can be used to predict cognitive decline and certain mental disorders like mild cognitive impairment, Alzheimer's disease, or dementia [49, 50].

On the other hand, some studies indicate that impairments in IADLs exhibit dynamic trends in the population, suggesting that interventions can enhance the performance of IADLs and promote prolonged independent living and an improved quality of life [48]. Meeting the increasing need for these interventions requires interdisciplinary collaboration among geriatric clinicians, rehabilitation specialists, and nurses specializing in older adult care. Designing interventions for the complex nature of BADLs and IADLs poses numerous psychological and sociological challenges when applied on a large scale among older adults. Notable challenges include data security and privacy, trust in digital tools, acceptance, ease of access and use, the need for training, adaptation time, potential loss of dignity, and issues related to social inclusion [25, 51, 52].

In this review, we have provided evaluation metrics that are used for the evaluation of interventions and technologies in their studies. Evaluation metrics are quantitative measures used to assess the performance and effectiveness of a system, model, or algorithm. These metrics provide objective and reliable methods for evaluating the quality and accuracy of the results produced. By incorporating the subjective viewpoints of users, this type of analysis provides insights into how well a system meets user expectations, satisfaction levels, ease of use, and overall effectiveness in solving the intended problems.

In order to assess the conceptual technologies' applicability, we followed the same approach of Morrison et al. [53] for the classification of effectiveness levels. Technologies were analysed to uncover any supplementary design elements that were not initially identified during the review of the diverse sample. Subsequently, the relationship between the presence of these design elements and the impact of the technologies was evaluated. The technologies within the representative sample were also categorized as more effective, less effective, or ineffective based on the results of evaluation metrics.

### Ambition and Aim of This Scoping Review

In the recent years, many scoping and systematic reviews have focused on interventions to improve the ADLs for older adults and effectiveness of different interventions used in home environments. While many of the studies focused on physical activities and exercise training for older adults to improve the quality of life [54, 55, 56, 57], the impact of interventions on those physical activities was only studied by Beswick et al. [58]. However, this study didn’t include the cognitive and physical interventions. The first studies for identifying the different types of interventions for ADLs and assessing their effectiveness were achieved by Motamed-Jahromi, M., and Kaveh [59] and Pajalic et al. [60]. Motamed-Jahromi, M., and Kaveh investigated eight studies and categorized the interventions into three different types: cognitive training, physical exercises, and multicomponent interventions [59]. Pajalic et al. included twelve studies by using risk of bias assessment method [60]. They showed that each technology should be considered according to needs of older adults, conditions of the environment and abilities of the participants. Similar recommendations were investigated and examined for different assistive technologies for older adults by Fotteler et al. [61]. More specifically, Kim et al. reviewed 30 studies and classified the smart home technologies and their architecture used for monitoring older adults in their homes [16].

Besides previous reviews, according to our knowledge, our review is the first study aiming at investigating (1) the different technological solutions according to different domains of IADL, (2) the used technological elements and software/algorithms, and their accessibility feature as open or close source, and (3) the evaluation tools with their results (4) the evaluation metrics and effectiveness analysis according to those metrics.

## 2. MATERIALS AND METHODS

### 2.1 Overview

This scoping review was prepared according to the six-stage methodological framework for scoping reviews outlined by Arksey and O’Malley [62] and PRISMA-ScR (Preferred Reporting Items for Systematic Reviews and Meta-Analyses Extension for Scoping Reviews) guidelines [63]. We followed those main steps of methodological framework: specifying the research questions; constructing the search strategy and finding relevant studies; selecting the relevant studies according to the predefined criteria; analysing the final list of the studies and charting the data; performing the collation, summarization, and reporting the results; then, finally, consultation the study findings with authors. PRISMA-ScR guidelines were used for guaranteeing our scoping review to be systematic, transparent, and complete.

### 2.2 Research Questions

Guidance of our scoping review was performed by these research questions inferred from the aim of the review:

What kind of technological solutions exits for specific domains of IADL?

1. What kind of technological tools/software are used and which of them have open source?
2. Which evaluation tools are used for usability/satisfaction/acceptance evaluation and what are the evaluation results?
3. What are the evaluation metrics of technological solutions for health and effectiveness?

### 2.3 Search Strategy

**Figure 1.**
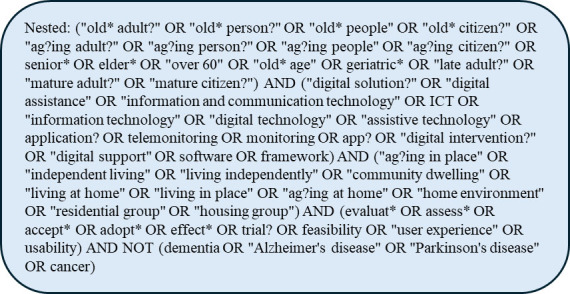
List of words and phrases used in search strategy.

### 2.4. Study Selection

**Figure 2.**
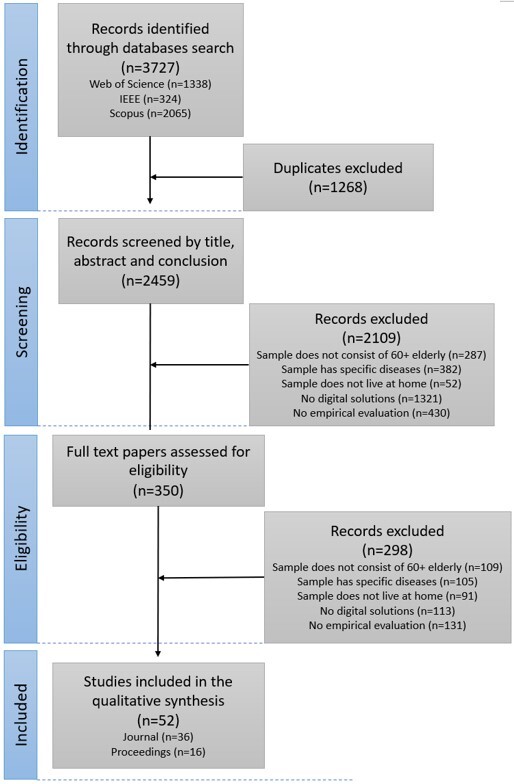
Flowchart of the search and study selection process.

We identified 3727 records in three databases (SCOPUS, WOS, IEEE) by screening the titles and abstracts of those records (Figure 2). After the removal of duplication studies, 2459 studies remained for screening phase. Six exclusion criteria were determined for the review in the screening phase. If one of the criteria was satisfied, that study was excluded from the review. Exclusion criteria for screening phase were:

Sample does not consist of 60+ older adults: only studies with samples consisting of older adults (I.e., those aged 60 or above) were included in the next stage.

Sample has specific diseases/issues, such as dementia: only studies with generally healthy older adults’ samples were included in the next stage. We did not want studies with specific samples, such as those struggling with dementia, Alzheimer's disease, Parkinson's disease, cancer, disabilities. Our assumption for the studies including specific studies is that those technological solutions are focusing on the issues of the specific cohort rather than the generally healthy older adults.

Sample is not community dwelling older adults: only studies with community dwelling older adults or housing groups were included in the next stage. We did not want studies conducted among older adults living in retirement homes etc.

No digital solutions: only studies with digital interventions were included in the next stage. If the focus is not on digital solutions that support community dwelling older adults, but rather on testing drugs, off-line psychological interventions etc., the study was not relevant for our review.

No empirical evaluation: only studies with empirical data on feasibility/effectiveness of digital solutions went to the next stage. If digital solutions were only described, but not tested, the study was not included. If digital solutions were only tested in a very technical sense (e.g., simulations), the study was also not relevant for us. Data need to be gathered on human participants.

All authors participated to the screening phase (UA, US, NP, BM and IM) by reviewing independently the titles and abstracts of the studies. Disagreements were settled through discussion between authors. At the end of the screening phase, 2109 studies were not fulfilling the criterion or more criterion. We noticed that some of the studies had more than one exclusion criteria. Thus, the total number of excluded records is lower than the summation of each criterion (n=2472) (Fig. 2).

The detailed assessment was performed in this eligibility phase by reading full texts of remaining all 350 studies by all authors. We tried to include only those articles that were relevant, so we needed to be much stricter than in the previous stage. Same exclusion criteria were applied as previous step (Fig. 2).

After the eligibility phase, each record (n=52) analysed by different two authors: Author 1 collected data regarding sample sizes, technology, and classify whether the study targeted IADL or BADL (if the study targeted IADL, further info was needed to note which specific area was targeted). Author 2 just needed to classify whether the study targeted IADL or BADL (if the study targeted IADL, further info was needed to note which specific area was targeted). This allowed us to check the classification agreement. All inconsistencies were discussed and solved in this step.

### 2.5 Data collection and Charting

In this step, specific information was extracted from final list of the studies by all authors to a spreadsheet. Parameters of each study added for the analysis were:

- **Sample size (older adults):** added info regarding the sample size of older adults.
- **Is technology described?:** added if technology used is described (if the article explains which technology was used to deliver the intervention).
- **If tech. described -> Architecture available?:** added if there was an image describing the system architecture or the conceptual model of technology is described in detail in the text (e.g., structure of technology, components).
- **Technology evaluation (UX, effects, … ) available?:** this was an additional check that technology was, indeed, evaluated by human participants. For example, was there info on user experience/system usability available? Or info regarding the effects of technology on any outcomes? Added if technology was evaluated.
- **IADL or BADL:** Added as IADL if the article targets IADLs, add as BADL if the article targets BADLs, add IADL/BADL if the article targets both.
- **Detailed IADL classification:** if the article targets IADLs, added info regarding which specific areas of IADLs were targeted. 1) finance (e.g. online shopping, managing money, paying the bills), 2) leisure activities (e.g., being included in leisure activities, entertainment, … ), 3) communication (e.g., using ICT, keeping in contact with relatives, video calling), 4) health (e.g., access to physicians, taking medications, physical activity), 5) mobility (e.g., organizing travel, running errands, … ), 6) home (e.g., cleaning, energy management), 7) food (e.g., ordering food, finding recipes, healthy diet).

### 2.6 Collating, Summarizing, and Reporting Results

All results were prepared and charted by UA and reviewed by the rest of the authors for maintaining the rigor in this step.

## 3. RESULTS

### 3.1 Study Design of IADLs Studies

After selection process of literature review, 52 studies remained in final list (see Table 1). All remaining studies were analysed according to 7 different IADLs. Most of the studies (n=36) targeted one IADL in the study design, some studies targeted more than one IADLs (5 of them targeted three IADLs and 11 of them targeted two IADLs). Most of the IADLs categories are health related activities (n=45, 60.8%) and communication activities (n=20, 27%).

**Table 1:** Final list of reviewed studies (IADL Class: 2=leisure activities, 3=communication, 4=health, 5=mobility, 6=home, 7=food; Subjective Effectiveness Score: 1= More effective, 2= Less effective, 3= Ineffective).

No	Study	Study Design	Study Duration	Sampling Size	Technology	Type Of Technology	Technology Evaluation Tools	Results of Technology evaluation Tools	IADL Class	Involvement of Older Adults to Implementation	Used Metrics for Effectiveness Evaluation	Subjective Effectiveness Score
**1**	Ballesteros et al. [70]	Not Specified	1 year	57	Social media	Web-Based Social Network Platform	Interviews	Qualitative data, impressions and opinions	3,4	Yes, continuous contact and taking advice from older adults for making the technology suitable for them.	Wellbeing (Affection, Assertivity, Status, Comfort, Stimulation)	2
**2**	Bevilacqua et al. [67]	Observational Study	Not Specified	67	Robot	A Companion Robot	TAL, SUS	TAL has produced not relevant information, mainly in the case of the Italian sample. SUS results of the Italian sample: Not usable (18%), M = 50.8; SD = ± 7.8, Usable (22%), M = 77.8; SD = ± 6.7 and Excellent (60%), M = 90.71; SD = ± 4). Swedish sample: Not usable (56%), M = 36.7; SD = ± 19.6, Usable (33%), M = 78.3; SD = ± 4.6 and Excellent (11%), M = 87.5; SD = ± 3.5.	3,4,5	No	Experience, Usability, Users’ Performance, Acceptance	2
**3**	Caine et al. [68]	Between Subjects Design	Not Specified	18	Robot	A Companion Robot	Interviews	Qualitative data, impressions and opinions	3	No	General Comfort, Perceptions, Reactions	2
**4**	Caroux et al. [82]	Field Study	9 months	13	Social media	Email Application	Customized	The effectiveness score for performing the timed-usage scenario increases across time (F(2,24) = 7.7; p = .003). Conjointly, a strong enhancement of efficiency score is observed (F(2,24) = 7.9; p = .002).	3,4	No	Comfort Level, Usability	2
**5**	Chi et al. [88]	Not Specified	3 months	10	Interactive	Tabled-Based ECA System	Interviews	Strongly dislike 10% (1), Dislike 10% (1), Neutral 20% (2), Like 50% (5), Strongly like 10% (1)	3,4	No	Perceived Difficulty, Usability, Satisfaction, Ease of Use	2
**6**	Chung et al. [95]	Not Specified	1-week	41	Mobile	Smart Phone App	USE	USE: 2.3 (0.56)	4	No	Retention, Adherence, Adverse Events, Physical Activity Enjoyment, Usability, Perceptions	1
**7**	Daly et al. [71]	Prospective Single-Arm Pilot Study	8-week	20	Home healthcare	Home-Based Exercise Program Via Tablet	SUS	SUS: 86 [SD:10]	4	No	Adherence, Feasibility, Adverse Events, Acceptance	1
**8**	Domingos et al., 2022 [126]	Observational Study	2 weeks	110	Mobile Technologies	Wearable Devices	SUS, USE	Mean user acceptance was very high—perceived ease of use: 6.45 (SD 0.78); perceptions of external control: 6.74 (SD 0.55); computer anxiety: 6.85 (SD 0.47); and behavioural intention: 6.60 (SD 0.97). The usability was excellent with an average score of 92.70 (SD 10.73)	4	No	usability, satisfaction	1
**9**	Fyfe et al. [75]	Randomized Clinical Trial	4 weeks	38	Home healthcare	Remote Monitoring And On-Line Training Package	Customized	39%, “a great deal”; 36%, “a lot”; 18%, “a moderate amount”; 4%, “a little”; 0%, “not at all”. Satisfaction from intervention: 79%.	4	No	Adherence, Feasibility, Ease of Use, Frequency Of Contact, Trust	1
**10**	Geraedts et al. [72]	Prospective Cohort Study	6-month	21	Home healthcare	Home-Based Exercise Program	Customized	The average score: 4.2 (SD: 0.2).	3,4	No	Experience	1
**11**	Göransson et al. [64]	Explorative Qualitative Design.	3 months	17	Mobile	Smart Phone App	Interviews	Qualitative data, impressions and opinions	3,4	No	Acceptance	1
**12**	Gross et al. [65]	Explorative Case Study	1 month	9	Robot	A Companion Robot	Interviews	Qualitative data, impressions, and opinions	2,3,4	No	Mobility, Usability, Enjoyment, User Acceptance	2
**13**	Gschwind et al. [76]	Randomized Clinical Trial	16 weeks	153	Home healthcare	Exergame And Fall Risk Assessment	SUS, PACES	SUS: 62 (SD = 23), PACES: 31 (SD = 8)	4	No	Familiarity With Technology, Usability, Acceptability	1
**14**	Hill et al. [80]	Descriptive, Mixed-Methods Design	2-hour training and practice session	9	Mobile	Smart Phone App	Interview (0-10)	Satisfying to use with an average rating of 8.2 (SD = 1.9), high in likeability (M = 8.5, SD = 1.6), interest (M = 8.8, SD = 2.3)	4	No	Adherence, Health-Related Quality Of Life, Usability	1
**15**	Hong and Lee, 2023 [127]	RCT	8 weeks	60	Home health technologies	Computerized program for well-being via tablet	Mobile App Rating Scale (MARS)	Total uMARS score average was 4.1 (SD = 0.7)	2,3,4	No	aesthetics, engagement, usability	1
**16**	Jansons et al. [73]	Prospective Single-Arm Feasibility Study.	12 weeks	15	Home healthcare	Home-Based Exercise Program	SUS	The mean SUS: 75 ± 19.	4	No	System Usage, Technical Issues	2
**17**	Kim et al., 2023 [128]			25	safety monitoring	sensors	UEQ	A total of 95% (19/20) of the items were answered over a mean of 1.6 (SD 1.19)	4	No	accessibility and intention to use.	2
**18**	Kurillo et al. [123]	Not Specified	18-week	6	Home healthcare	Exergame And Fall Risk Assessment	Interviews	the subjects who did use the system on regular basis provided mostly positive impressions.	4	Yes, first prototype deployment was designed with participants.	Usefulness, Ease of Use, Satisfaction	1
**19**	Lee et al. [85]	Long-Term Field Trial	8 weeks	4	Home healthcare	Medication Management Technology	Interviews	Qualitative data, impressions and opinions	3,4	No	Time Of Access, Duration, Score, Usability, Technological Equivalence, Usage Time, Satisfaction	1
**20**	Luperto et al. [83]	Preliminary Usability Test	10 weeks	25	Home healthcare	Computerized Program For Well-Being Via Tablet	Customized	Median: 4, Positive use of the system: 72.7%, Satisfaction: 68.2%.	2,3,4	Yes, suggestions about activities and scenarios were taken from older adults.	Acceptance	1
**21**	Luperto et al. [87]	Experimental Campaign	6 months	13	Robot	A Companion Robot	Customized	Mean: 3. 6, IQ: 2.8	3,4	No	*not yet published	
**22**	Macis et al. [90]	Not Specified	4 months	7	Telehealth	Telemonitoring	Interviews	Not yet published	3	No	Attitudes Toward Smartphones, Perceived Difficulty, Acceptability, Usability, Application Use	1
**23**	Mahlo and Windsor [96]	Not Specified	30-day	46	Mobile	Smart Phone App	ATCQ	ATCQ: 0.9, 0.8, and 0.7 for the Comfort, Efficacy, and Interest subscales, respectively	4	No	Usability, Task Completion, Task Efficiency, Error Rate, Task Perceived Difficulty	1
**24**	Moral et al., 2023 [129]	participatory design	6 months	48	Mobile Technologies	mobile app	SUS	mean 90.00 (6.96)	4	yes, co-creation approach was followed for design, development and evaluation phases.	usability	1
**25**	Nguyen et al. [100]	Not Specified	1-month	12	Mobile	Smart Phone App	SUS	Occupational therapists (n=6): mean SUS: 91.7 (8.0), Older adults (n=6): mean SUS: 71.7 (27.1).	6	No	Acceptability, Needs for Education	2
**26**	Noury et al. [98]	Usability Testing	6 months	12	Safety Monitoring	Sensors	Interviews	qualitative data, impressions, and opinions	4	No	Use Of the System, Effective Exercise Rate, Successfully Completed Exercises	1
**27**	Ofli et al. [99]	Not Specified	6 weeks	6	Interactive	Online Coaching	Customized	Effective system usage: 76%	4	No	Use Of the System, Activity Levels, Integration into Daily Routines, User Preferences, Health-Related Quality Of Life, Increased Activity And Social Engagement, Improvement Of Strength And Balance, Technology Access	1
**28**	Ogonowski et al. [124]	Not Specified	6-month	12	Home healthcare	Exergame And Fall Risk Assessment	Interviews	Qualitative data, impressions and opinions	4	Yes, a Living Lab approach was used.	Usability, Attitude, Experiences	1
**29**	Oppert et al., 2023 [130]		8 weeks	10	Home health technologies	Remote monitoring and On-line training package	interviews	Participating in virtual tour experiences was well accepted as participants expressed enjoyment, nostalgia, and interest in future use	2	No	accessibility and intention to use.	2
**30**	Panagopoulos et al. [92]	Not Specified	Not Specified	26	Home healthcare	Computerized Health Monitoring Program	SUS	SUS score: average = 75, median = 72.5 for female, average = 70.7, median = 72.5 for male respondents. The overall score is 72.8.	4	No	Acceptance, Usability	1
**31**	Parker et al. [86]	Case Study	8 weeks	31	Home healthcare	Medication Management Technology	Customized	Helpful or very helpful: 77%, Acceptable or Very acceptable: 74%, Easy or Very easy to use: 100%	3,4	No	*not yet published	
**32**	Perotti and Strutz, 2023			106	robot technologies	an assistive robot	Technology Usage Inventory (TUI)	The acceptance, usability and usefulness of the system were rated as medium, while the intention to use was rated as low.	7	no	usefulness, usability, accessibility and intention to use.	2
**33**	Piau et al. [74]	Pilot Study	6 months	25	Safety Monitoring	Sensors	Customized	not yet published	4	No	Utility, Acceptability	2
**34**	Pigini et al. [93]	Prospective Open-Label Study	Not Specified	30	Robot	A Companion Robot	Interviews	Monitoring the situation 4,6 (0,8, Emergency intervention 4,5 (0,8), Standing up assistance 3,8 (1,3), Fetch and carry 3,6 (1,4), Preparing food 2,4 (1,4)	4	No	Usability, Benefits	1
**35**	Pripfl et al. [94]	Not Specified	3 weeks	7	Robot	A Companion Robot	NARS	General attitude towards robots did not change for the two subscales “negative attitude towards interactions with robots and social influence of robots”; however, the third subscale “negative attitude towards emotions in interaction with robots” showed a tendency towards significance (p = 0.089).	4	No	Attitude, Safety	2
**36**	Ravindran et al., 2023	Cohort Study	2 weeks	35	safety monitoring	sensors	Customized acceptability questionnaire	Very easy to use	4	No	Acceptance	1
**37**	Ray et al. [69]	Not Specified	2 months	50	Home healthcare	Remote Monitoring and On-Line Training Package	Interviews	Qualitative data, impressions and opinions	3	Yes, continuous engagement was provided between the researchers and various stakeholders.	Usefulness, Attitude, Ease of Learning, Design of Interface, Ease of Use	1
**38**	Reynolds et al., 2023 [133]	longitudinal, observational, prospective pilot study	12 weeks	16	safety monitoring	sensors	survey	all participants reporting “Agree” or “Strongly Agree” across all questions	4	no	acceptability	1
**39**	Shahid et al., 2022 [134]	experimental setup	1 year	12	safety monitoring	sensors	Customized questionnaires for the use of system (Likert 5)	mean=4	4	yes, interview was used co-creation process	user satisfaction with the system	1
**40**	Silveira et al. [97]	Comparative Case Study	2 weeks	13	Mobile	Smart Phone App	Customized	Navigate through the messages posted on the Bulletin Board (91%); read the posts from the Bulletin Board (100%); and receive messages on the inBox (73%).	4	No	Usability, Attrition, Adherence, Effectiveness	1
**41**	Smith et al. [102]	Not Specified	8 weeks	7	Home healthcare	Exergame And Fall Risk Assessment	Interviews	Qualitative data, impressions, and opinions	4	No	Use The System	2
**42**	Teixeira-Santos et al. [81]	Not Specified	10 days	96	Safety Monitoring	Sensors	Interviews	Easy to use: 95.8%, some discomfort: 33.7%, no changes in daily routines: 91.7%, sensitized to adopt healthy lifestyles: 56.3%, data obtained through sensors are useful to improve their health status: 93.8%.	4	No	Ease Of Use, Functionality, Perception of The Usefulness	1
**43**	Ting and Lewkowicz [89]	A Mixed-Methods Study	one month	6	Interactive	Embodied Interactional Practices On A Full Device (TV Monitor, PC, Kinect)	Interviews	Confirmed the usefulness of the services and the interest of multimodal interaction	3	No	Usefulness, Acceptability	1
**44**	Tirkel et al. [125]	Not Specified	Not Specified	6	Home healthcare	Home-Based Exercise Program	Interviews	All the participants responded positively to the question whether they use the computer or the television, most of them used a computer for several hours a day.	4	No	Preference	1
**45**	Tsiourti et al. [66]	Observational Study	12 weeks	20	Interactive	Autonomous Conversational Agent System	SUS	Average SUS: 62.2 for Switzerland and 52 for the Netherlands. The combined SUS score: 58.8.	3	Yes, user-centred design studies were applied.	Acceptance, Usability, Usefulness, Level Of Autonomy, Health-Related Quality Of Life, Impact On Their Daily Life, Effectiveness, Efficiency, Satisfaction	2
**46**	van Doorn-van Atten et al. [77]	Exploratory Evaluation Study	3-month	20	Telehealth	Telemonitoring	Interviews	The majority of participants found it easy to weigh themselves (89%), to use the pedometer (70%), to use the sphygmomanometer (100%), and to use the tablet (67%)	4,7	No	Reach, Fidelity, Acceptability, Effect Measurements	1
**47**	Vaziri et al. [78]	Randomized Clinical Trial	16 weeks	153	Home healthcare	Exergame And Fall Risk Assessment	SUS, PACES, DART	SUS: 62 (SD 15.58); PACES: 31 (SD 8.03); DART:0.87 (SD 0.26)	4	No	Usability, Experience, Acceptance	2
**48**	Vaziri et al. [79]	Randomized Clinical Trial	16 weeks	153	Home healthcare	Exergame And Fall Risk Assessment	Interviews	Qualitative data, impressions and opinions	3,4	No	Benefits, Problems, Usage, Level Of Difficulty, Easy To Interact, Error Tolerance	1
**49**	Wang et al. [91]	Multisited two-group randomized study	3-month	45	Telehealth	Telemonitoring	Customized	Using the system is a good idea: mean 5.7, SD 1.1, System is comfortable: mean 4.9, SD 1.1, Useful to improve their health: mean 5, SD 0.9, Easy to use: mean 5.0, SD 1.0, Positive intention to use in their future health management: mean 4.4, SD 1.8.	3,4	No	Usefulness, Acceptance, Self-Efficacy, Ease Of Use,	1
**50**	Ye et al., 2022 [135]	RCT	8 weeks	59	Mobile Technologies	Mobile app	Customized questionnaires for the use of system (Likert 5)	The majority of older adults (78.81%) provided positive evaluations	4	No	Satisfaction	1
**51**	Zhao et al., 2023 [136]			44	Safety monitoring	Sensors	Customized questionnaires for the use of system (Likert 7)	Good idea (mean = 5.45, SD = 0.76), intention to use: mean = 5.22, SD = 1.10 and easy (mean = 4.95, SD = 1.09)	4	no	Trust, acceptance, usability	2
**52**	Zsiga et al. [84]	Follow-up study	3 months	8	Robot	A Companion Robot	Customized	The mean score for all services: 3.9 for usefulness, 3.7 for reliability, and 4.4 for satisfaction.	2,3,4	No	Usefulness, Reliability, Satisfaction, Number of Interactions	1

As expected, case studies from descriptive studies are the most preferred study design (n=29) to evaluate the technology for older adults. Between case studies, three studies [64, 65, 66] described their study as explorative case study and other three studies [67, 68, 69] described as comparative case study. These case studies, in general, evaluate the experience, usability, satisfaction and acceptance with the patient’s symptoms, signs, diagnosis, and treatment. For the rest of the studies: five studies are designed as cohort studies to compare different groups of patients (with/without control) [70, 71, 72, 73, 74, 132]; five studies performed randomized clinical trials [75, 76, 77, 78, 79, 127, 135] and only two studies used mixed-methods study [80, 81].

According to the study duration, the IADLs studies performed mostly between 1 - 12 weeks period (63.4%). Only six of them performed between 12 - 23 weeks (13.8%) and 23 - 34 weeks (11.5%). There is only one study for long term evaluation between 34 - 45 weeks [82] and two for 45 - 56 weeks [70, 134]. Five studies did not provide the duration information for their studies.

Most of the studies had participants between 4 and 44 (73%). Eigth studies performed with 44 to 84 participants; three studies had participants between 84 and 124, and only three studies, which are RCT studies, had between 124 and 164 participants. As expected, the types of study designs were the main factor that affecting the sample size results. The number of participants is increasing from the case study designs to RCT designs in general.

### 3.2 Technologies/Software Tools in Reviewed IADLs Studies

In our review, technology types in IADLs studies were investigated. The most preferred three technologies for the older adults in IADLs studies are home healthcare technologies (n=23, 44.2%), robot technologies (n=8, 15.4%) and mobile technologies (n=9, 17.3%). The details of usage of each technology type according to IADL categories are given in the following subsections (see Table 1).

#### 3.2.1 Leisure Activities:

Five studies from the list of remaining studies were in the IADL category of leisure activities. Three of them were using *home healthcare technologies* [83, 127, 130] for leisure activities for older adults, and other two studies offered *robot technologies* [65, 84] to older adults. For home healthcare technologies, computerized programs via tablet and remote monitoring were provided to participants to promote active well-being for the older adults. For the studies with robot technologies, companion robots were used for keeping older adults physically and mentally fit in their home environments.

#### 3.2.2. Communication Activities:

Communication activities are the second most targeted IADLs in remaining studies (n=20). For this type of IADLs category, 35% (n=7) studies were for *home healthcare technologies*, 25% (n=5) for *robot technologies*, 15% (n=3) for *interactive technologies*, 10% (n=2) for both *social media technologies* and *telehealth technologies*, and 5% (n=1) for *mobile technologies*.

Two studies which used home healthcare technologies [85, 86] performed medication management technology for medication reminder and medication monitoring purposes. The remaining five studies with home healthcare technologies [69, 72, 79, 87, 127] performed via online exercise or software programs with the aim of promoting well-being and reducing fall risks.

All studies with robot technology used the companion robots to provide different improvements to older adults such as cognitive assistance [84], domestic health assistance for independent living [65, 67], enhancing behaviors [68] and daily monitoring [87].

For the usage of interactive technologies, all three studies [66, 88, 89] used embodied conversational agents for a reminder, guidance and social communication.

Web-based social network platform [70] and email application [82] were used in studies with social media technologies by trying to increase the social interactions and communication behaviours of older adults.

The studies used telehealth technologies [90, 91] collected information via telemonitoring to record the health-related information in a user-friendly platform.

Only one study [64] used mobile technologies to examine the home-based health care experiences for older adults.

#### 3.2.3. Health Related Activities:

Health related activities are the most targeted IADLs between remaining studies (n=45) according to our literature review. For this type of IADL category, 37.8% (n=17) studies used *home healthcare technologies*, 13.3% (n=6) *robot technologies*, 17.8% (n=8) *mobile technologies*, 17.8% (n=8) *safety monitoring*, and 4.4% (n=2) *social media technologies, interactive technologies, and telehealth technologies*.

Most of the studies which used home healthcare technologies performed exercise programs/exergames and fall risk assessment (n=10) for older adults. The common purpose of exercise programs/exergames studies is to investigate the fall risk factors, to reduce the fall and to evaluate digital exercise solutions for older adults in their homes. Online monitoring programs were second most used approach (n=5) in home healthcare technologies [69, 75, 87, 92, 127]. Improving communication skills of older adults and remote monitoring for health factors are the main purposes of the studies. And remaining studies (n=2) [85, 86] performed medication management technology to monitor and to remind the medicines to older adults.

In the studies with robot technologies (n=6), most of the studies (n=3) used robots as a monitoring tool to collect health information [67, 87, 93]. On the other hand, two studies provided cognitive assistance [65, 84] and one study detected fall risks [94] for older adults via companion robots.

Studies with mobile technologies (n=8) targeted different purposes such as application usage evaluation [64, 95], attention training [80], meditation [96] and physical training [97, 135, 129, 126].

All studies with safety monitoring (n=8) used sensors for monitoring and follow up the older adults for any dangerous cases [81, 98, 128], sleep monitoring [132, 133], fall detection [136] and producing alerts [74, 134].

Studies with interactive technologies (n=2) used online coaching [99] and embodied conversational agents [88] to evaluate the performance of daily activities of older adults.

Studies with telehealth technologies (n=2) performed telemonitoring for collecting health-related information [91] and improving the nutritional status [77] of the older adults.

Two studies used social media technologies [70, 82] to improve the active lifestyle and communication of older adults.

#### 3.2.4. Mobility Activities:

Mobility activities were targeted only by Bevilacqua et al. [67] by using robot technology to provide intelligent environment by different integrated services for independent older adults living. Although Bevilacqua et al. offers a robot platform which has different mobility features such as shopping, food delivery, garbage collection and walking assistance, they reported results only for shopping as a mobility activity [67].

#### 3.2.5. Basic Home Activities:

Only one study was performed by Nguyen et al. targeting basic home activities via smart phone applications [100]. In this study, home modifications were tested via mobile application with the help of occupational therapists. Those home modifications included main replacement of things in the home to remove the barriers that can have a potential for injuries or falls for older adults.

#### 3.2.6. Food Related Activities:

Food related activities are assessed via telemonitoring system to enhance the nutritional level of the older adults [77] and interactive robot arms for food preparation [131]. Body weight control, diet quality, blood pressure and additional nutrition scores were evaluated.

### 3.3. Evaluation Tools of Technologies and Evaluation Results for IADLs

In our review, we investigated the evaluation tools which were used to assess the usage, acceptance, and other factors of the different technologies, again, according to IADLs categories. In remaining studies, interview was the most preferred method for the evaluation of technologies in IADLS studies. System usability scales (SUS) and customized questionnaire (both for use of system and acceptance) tools were the second most used in IADLs studies.

#### 3.3.1. Leisure Activities:

Studies targeted leisure activities used *customized questionnaires for the use of system* [83, 84], Mobile App Rating Scale (MARS) [127] and *open-ended interview* [65, 130] for the evaluation of technologies for older adults. All studies got meaningful and satisfied results. There is no statistics or numerical evaluation metric from open-ended interview studies, they include only opinions (users’ appreciation and emotional binding in a positive way) of the users in a narrative way.

#### 3.3.2. Communication Activities:

For this IADL category, six different technology evaluation tools were used in 20 studies (*open-ended interview* (50%), *customized questionnaire for use of system* (25%), *customized acceptance questionnaire* (15%), SUS (5%), MARS [127] (5%) and *thinking aloud technique* (TAL) (5%)).

Between studies with open-ended interview evaluation, eight studies [64, 65, 68, 69, 70, 79, 85, 89] provided impressions and opinions in a narrative way (positive attitudes towards tools and usefulness of the services), Chi et al. provided evaluation results as percentages [88], and Macis et al. stated that they are still in testing phase and the results have not published yet [90].

Studies with customized questionnaire for use of system used different scales: three of them [72, 83, 84] showed results in Likert scale; Caroux et al. gave only effectiveness and efficiency scores [82].

Similar different scale usages were observed in studies with customized acceptance questionnaire: percentages [86], Likert scale [91], and Mean scores [83].

MARS was used when there was a limitation at coronavirus disease times for the remote evaluation of interventions [127]. TAL and SUS were used for mobility activities by Bevilacqua et al. to evaluate the robot technology [67]. Relevant information could not get from TAL scale; however, SUS gave better results for Italian sample than Swedish sample. SUS was also reported by Tsiourti et al. [66] with the combined SUS score.

#### 3.3.3. Health Related Activities:

45 studies evaluated the technologies via thirteen different tools. The percentages of the tools are: *open-ended interview* (34%), *customized questionnaire for use of system* (23.4%), *SUS* (17%), *customized acceptance questionnaire* (6.4%), *Physical Activity Enjoyment Scale (PACES)* (4.3%), The Attitudes Towards Computers Questionnaire (*ATCQ)* (2.1%), *Negative Attitude toward Robots Scale* (*NARS)* (2.1%), *dynamic acceptance model for the re-evaluation of technologies (DART)* (2.1%), MARS (2.1%), UEQ (2.1%) and *Usefulness, Satisfaction, and Ease of Use (USE)* (2.1%). Like for communication activities, the most used evaluation tool for health-related activities is interview. While ten studies express the impressions and opinions of participants in a narrative way and did not provide any numerical results, the remaining five studies reported some metrics such as: ‘satisfying rate’, ‘likeability’, ‘interest’ [80]; ‘likability’ [88]; ‘usability’ [77]; ‘user-friendliness’ [81]; ‘different levels of active involvement’ [93].

Each study that used customized questionnaire for use of system reported different scales and metrics within their publications: effective system usage [99]; performance of the timed-usage scenario and efficiency score [82]; the results for navigation, reading the posts, and receiving messages [97]; the positive use of the system and satisfaction [83]; only mean score from Likert type scale [72]; satisfaction from intervention [75]; mean score for all services for usefulness, for reliability, and for satisfaction [84]; and not publishing any results of evaluation because of continuation of trials [74].

SUS is the third most used evaluation tool for category 4. Six studies performed SUS and reported different SUS scores [67, 71, 73, 76, 78, 92].

Customized questionnaire for acceptance was used by two studies [86, 91]. Parker et al. reported the results in percentages [86] and Wang et al. used Likert type scale for the results of customized questionnaire for acceptance [91].

For the studies with PACES evaluation tool, two studies reported the PACES scores in their studies [76, 78].

On the other hand, each of ATCQ, MARS, UEQ and NARS evaluation tools were used only by one study.

#### 3.3.4. Mobility Activities:

TAL and SUS were used for mobility activities by Bevilacqua et al. [67] for this category. Relevant information could not get from TAL scale; however, SUS gave meaningful results.

#### 3.3.5. Basic Home Activities:

SUS was used as an evaluation tool for the study targeting basic home activities [100] via smart phone applications.

#### 3.3.6. Food Related Activities:

Food related activities were evaluated with open-ended interview by van Doorn-van Atten et al. [77] and Technology Usage Inventory (TUI) by Perotti and Strutz, 2023 [131]. Participants commented positively on the usage of technologies for both studies.

### 3.4. Open-Source Availability

Among remaining studies, only 4 studies (7.7%) gave open-source information and/or github link about some of the digital components of their solutions. However, only Smith et al. provided its core codes as open source [102]. Tsiourti et al. provided repository info about an overview over all feature point algorithms implemented in Point Cloud Library (PCL) framework for object recognition in human behaviour and environment analysis (https://github.com/PointCloudLibrary/pcl/wiki) [66]. Also, same study provided the download option of character animation platform originally developed at the USC Institute for Creative Technologies (https://smartbody.ict.usc.edu/download2). Macis et al. provided information for the calendar application of the solution which is not so important within the whole solution (http://en.osdn.jp/projects/sfnet_biweekly/downloads/0.4.1/biweekly-0.4.1.jar/) [90]. Similar with previous studies, Luperto et al. provided the github repository for only one functionality (Deck of Cards) of the whole solution as open source (https://github.com/deck-of-cards/deck-of-cards) [83]. Only one study shared its development as an open source by Smith et al. (https://github.com/stepmania/stepmania) [102]. They developed a system called StepMania which is an advanced cross-platform rhythm game for home and arcade use (https://www.stepmania.com/).

### 3.5. Involvement of Older Adults to Implementation

In only 8 of the remaining studies (15.4%) were the technological solutions developed using the co-creation approach with the opinions of the participants. In most studies involving older adults, they were included in all phases of the study to make the technology suitable for them [70], to better understand the needs of older adults [123], and to incorporate their suggestions on activities and scenarios [83, 134].

### 3.6. Evaluation Metrics and Subjective Effectiveness Analysis

As seen from the Table 1, we identified 84 different evaluation metrics for the reviewed 52 papers. ‘usability’ and ‘acceptance’ are by far the most preferred evaluation metrics in our review. While the metric ‘usability’ is used in 34 studies (40.5%), the metric ‘acceptance’ is used in 18 studies (21.4%). The other most frequently used metrics are listed as follows: ‘ease of use’ (7 studies, 8.3%), ‘satisfaction’ (8 studies, 9.5%), ‘adherence’ (4 studies, 4.8%), ‘attitude’ (4 studies, 4.8%) and ‘experience’ (4 studies4.8%).

Morrison et al. has provided useful criteria for evaluating the effectiveness of e-health interventions and technologies [53], which we can utilize. Moreover, we have modified and expanded upon their approach to assess the outcomes of the technologies in older adult’s care, as illustrated in Table 1. According to the results, most of the technologies used in the reviewed studies got the ‘more effective’ score (33 studies, 63.5%) and ‘less effective’ score (17 studies, 32.7%). We could not evaluate 2 studies because of not having yet published results. None of the studies got ‘ineffective’ score in our review.

The percentages of subjective effectiveness scores for each technology were calculated as follows: home health technologies (total 17 studies, ‘more effective’: 73.3%, ‘less effective’: 26.7%), mobile technologies (total 9 studies, ‘more effective’: 88.9%, ‘less effective’: 11.1%), robot technologies (total 8 studies, ‘more effective’: 37.5%, ‘less effective’: 62.5%), interactive technologies (total 4 studies, ‘more effective’: 50%, ‘less effective’: 50%), telehealth technologies (total 2 studies, ‘more effective’: 100%), social media technologies (total 2 studies, ‘more effective’: 50%, ‘less effective’: 50%), safety monitoring (total 7 studies, ‘more effective’: 57.1%, ‘less effective’: 42.9%) and medication management technology (total 1 study, ‘more effective’: 100%).

## 4. DISCUSSION

### 4.1 Key Findings

The aim of this review was to identify recent technological solutions that monitor aging adults’ health and well-being in context of different IADLs, specify these technologies and their types used in the IADL studies and to provide comparison among the technologies with respect to evaluation tools results for usability/satisfaction/acceptance. Our main inclusion criteria for studies were having participants older than 60 years old, not targeting any specific disease, including community dwelling older adults, providing a digital solution for participants, and providing evaluation results. This scoping review identified 52 studies focusing on technological solutions for aging adults and investigated important factors such as: study design, intervention duration, sample size, technology, type of technology, technology evaluation tools and their results. In addition, remaining 52 studies were classified according to potential outcomes of the technologies such as social connectedness, autonomy and independence, mental health and wellbeing, physical health, and safety. Consequently, we aim to extend the existing research findings in the literature into the implications of the identified technological solutions for IADLs studies, types of those technologies and evaluation results of those technologies for community dwelling older adults.

#### Discussion on Study Design of the Studies

Most of the remaining studies in our review were observational studies (75%), as expected. They tried to search for the answers of usability, acceptance, and efficiency properties of the technological solutions via detailed and comprehensive description of cases with/without control group. Among these observational studies, two different study designs were used: case studies (63.5%) and cohort studies (11.5%). For other types, RCT study from experimental study type (13.5%) and mixed-methods study design (3.9%) were preferred.

#### Discussion on IADLs Categories of the Studies

Our analysis in context of IADL showed that the studies focused mostly on health (60.8%) and communication activities (27%) by providing a digital solution accompanying with its evaluation results for community dwelling older adults. Other IADL categories (finance, leisure, mobility, home, or food) could not find enough attention. This finding is in line with the recent literature about typical problems related with aging [101]. Among these problems, having problems with self-sufficiency, tending to accidents at home, having multiple health problems, increasing neurological disorders, feeling loneliness and in relation with this, increasing depression status are directly related to communication and health activities that have to be solved primarily for older adults. In studies with health IADL category, all types of technologies were used mainly focusing on home healthcare (n=17), robot (n=6) and mobile (n=8) technologies. On the other hand, studies with communication IADL category focused mostly on home healthcare (n=7) and robot (n=5) technologies.

#### Discussion on Technologies/Software Tools of the Studies

One of the main key findings of our review is about specifying the technologies used in the studies with respect to different IADLs. Although there are many types of technologies and naming for similar technologies in the literature, we categorized the technologies and their types in line with previous similar reviews [16, 25, 61, 101].

According to both number of studies (44.2%) and number of IADL categories (36.5%), the most preferred technology from our analysis are home healthcare technologies. Home healthcare technologies includes different types of technologies especially for well-being and prevention from health problems. Home healthcare technologies in the remaining reviewed studies were applied mainly for exercise programs, fall risk assessments, prevention of falls and medication management. Consequently, most of the studies with home healthcare technologies targeted IADL category of health (62.9%).

Robot technologies has the second highest usage rate in our review (15.4% of number of studies and 18.9% of number of IADL categories). Most of the studies with robot technologies targeted IADL category of health (42.8%) and IADL category of communication (35.7%).

Mobile technologies were also used in 17.3% of the studies and 14.86% of the IADLs categories. Interestingly, most of the studies with mobile technologies targeted IADL category of health (72.7%) rather than IADL category of communication.

The rest of the technology types have relatively minor usage in IADLs studies.

#### Discussion on Involvement of Older Adults to Implementation

Co-creation, the collaborative process of designing and implementing research studies and activities with the active involvement of older adults themselves, holds immense importance in ensuring that research findings and interventions are truly meaningful and impactful for this demographic. By directly engaging older adults in the design process, researchers gain valuable insights into their preferences, needs, and concerns, enabling them to develop studies and activities that are tailored to their specific circumstances, interests, and abilities. This participatory approach not only enhances the relevance and effectiveness of research but also fosters a more inclusive and empowering research environment that recognizes older adults as active partners in research rather than mere subjects. While the involvement of older adults in our review was relatively low, the impact of participatory design was evident in studies that employed this approach [69, 83, 123, 124, 129, 134]. These studies demonstrated higher effectiveness rates compared to those that did not involve older adults in the design process. These findings underscore the significance of a co-creation approach throughout the study design and implementation phases.

#### Discussion on Open-Source Availability

Open-source availability for digital solutions for the older adults is a significant problem for researchers in the field (only 1 study with open-source code and 4 studies with code links among 52 remaining studies). As society continues to witness a rapid growth in the aging population, the need for effective and user-friendly digital solutions for the older adults becomes crucial. Open-source software has played a significant role in facilitating innovation in various domains. However, the availability of open-source solutions specifically designed for the older adults is currently limited. One reason for this lack of open-source solutions is the relatively small market size. Compared to younger demographics, the elderly population may have lower adoption rates and requirements that are not catered to by mainstream technologies. Consequently, commercial software developers may consider the elderly market segment less lucrative, leading to fewer open-source initiatives. Another factor contributing to the non-availability of open-source digital solutions for the older adults is the complexity of designing inclusive and user-friendly interfaces. Developing software that accommodates the cognitive and physical challenges often associated with aging requires specialized expertise and resources. Additionally, accessibility and privacy concerns are critical considerations when developing digital solutions for the older adults. Ensuring compliance with accessibility guidelines and safeguarding user data can be challenging. Encouraging multidisciplinary research and fostering partnerships between academia and industry can foster innovation in this field. Furthermore, initiatives aimed at raising awareness and funding open-source projects catering to the older adults can incentivize developers to contribute their skills and expertise.

#### Discussion on Potential Outcomes of the Technologies

Besides all in-home technological developments and having wide range of application areas for older adults, some main issues such as selecting the right solutions, installing into home environment, and adapting to use for older adults are still valid and remain to be solved. For overcoming those issues, involvement of the older adults into the design of the technologies, plan of the intervention design and technology implementation in place is crucial to understand the effectiveness of the technological solutions. As stated in the introduction part, Rolfe et al. categorized the technological solutions for older adults according to their potential outcomes to identify the actual needs, confidence with technology and concerns at usage [39]. To get deeper understanding of the results of our findings, we further analysed the technologies in the remaining studies according to Rolfe et al. [39]. Results show that most of the studies reviewed in this study target health, wellbeing, independent living and physical exercise which is in line with our research questions and search strategies within this review. Social communication and safety outcomes have less interest in the remaining studies.

#### Discussion on Evaluation Tools and Their Results of the Studies

The remaining studies in review used wide variety of technology evaluation tools and the most preferred evaluation tool is doing interviews with participants via collecting qualitative data by taking their impressions and opinions (53.8%). Interviews conducted for program evaluation are typically qualitative but may also include some quantitative. Interviews may be useful from different perspectives such as to gather subjective perspectives from the participants, to evaluate individual differences between respondents’ experiences and outcomes, and to use as a follow-up to other evaluation tools. On the hand, being open to bias, requiring more time and effort, and having intrusive appearance to participants are the main issues with interviews [103].

Customization of questionnaires is used mainly because of two reasons. First, all the questions or modules in the standard questionnaire cannot be expected or recommended to be used for all countries or cases. Secondly, all variety of experiences around the world cannot be covered by a single questionnaire. Thus, some parts of the questionnaires are changed by making modifications, deletions, translation, or additions [104]. In the second place, customized questionnaires (for use of system and acceptance) take place (44.2%) as an evaluation tool for technology usage in our review. Most of the studies used the ‘5-point/7-point Likert scale’ and content validation for their adapted questionnaire.

SUS questionnaire is among the most preferred evaluation tool between the remaining studies (21.2%). SUS mainly measures the usability of digital tools in a subjective perspective [105]. It gives a result between 0-100. SUS has a wide popularity because of having perfect psychometric properties [106] and providing evaluation to any technological tool such as mobile applications, web portals, online training systems high validity and reliability [107].

Other evaluation tools used in the remaining studies were preferred a few times. Enjoyment of the older people was measured with Physical Activity Enjoyment Scale (PACES) by Kendzierski and DeCarlo [108]. It was designed as a unidimensional at the beginning, then it was improved by adding extra dimensions for different target groups [109, 110] and experiences [111, 112]. Usefulness, Satisfaction, and Ease of Use (USE) questionnaire was adopted version of SUS with a broad scope of application [113]. Because of having some issues related with reliability and validity, USE could not find a widely usage for the evaluation of technology usage [114]. Thinking aloud technique (TAL) is one of the simplest evaluation tools for usability [115]. Mainly, participants were asked to use the system continuously and to specify their thoughts verbally in a simple way. Besides having advantages such as being cheap, easy to learn and flexibility, unnatural situations, biasing and filtered statements can be mentioned as the main disadvantages of the tool [116]. Different factors in the communication with robots were analysed by Negative Attitude toward Robots Scale (NARS) [117]. This tool aims to measure the attitudes of the participants towards robot in their daily life. Its internal consistency and validity were confirmed by different studies [117, 118]. The Mobile App Rating Scale (MARS) stands out as a cutting-edge mHealth app quality assessment tool, meticulously evaluating apps across engagement, functionality, aesthetics, information accuracy, and overall subjective quality. Unlike its predecessors, MARS adopts a multifaceted approach, making it well-suited for assessing apps across diverse health domains [127]. Dynamic Acceptance Model for the Re-evaluation of Technologies (DART) was developed for the integration of users’ acceptance preferences into analysis of innovative solutions. DART includes four main dimensions: usefulness, ease of use, network effects and costs. Although some adaptation versions were released, it could not widely used in applications [119]. As a last tool, The Attitudes Towards Computers Questionnaire (ATCQ), was designed as 35-items scale to assess in seven dimensions of computer attitudes [120]. ATCQ used in several studies to measure the effect of online applications on older adults [121, 122].

#### Discussion on Evaluation Metrics and Subjective Effectiveness Analysis

The results presented in Table 1 highlight the prevalence of certain evaluation metrics in the 52 reviewed papers. Notably, the metrics of 'usability' and 'acceptance' emerge as the most frequently utilized in the studies. The prominence of these metrics suggests their significance in assessing the effectiveness and user satisfaction of the evaluated systems or technologies. Moreover, the analysis reveals several other commonly employed metrics, namely 'ease of use', 'satisfaction', 'adherence', 'attitude', and 'experience'. Although each of these metrics is observed in a smaller number of studies, their inclusion in multiple investigations underlines their relevance as valuable indicators of user experience and system performance. Overall, the prevalence of these evaluation metrics showcases the emphasis placed on user-centric factors and the need to comprehensively assess the usability and acceptance of technologies within the examined studies.

Based on the results of the review, it is evident that most of the technologies used in the reviewed studies were deemed 'more effective' and 'less effective'. Unfortunately, two studies were unable to be evaluated due to a lack of published results. Surprisingly, none of the studies were found to be 'ineffective' in our review. Furthermore, the percentages of subjective effectiveness scores were calculated for each specific technology. Home health technologies were the most common, with a total of 17 studies evaluated. These technologies received a 'more effective' rating in 70.6% of the cases, while 29.4% were deemed 'less effective'. Mobile technologies, consisting of nine studies, had a high percentage of 'more effective' scores at 88.9%, with only 11.1% being considered 'less effective'. Robot technologies, with eight studies evaluated, showed a relatively mixed effectiveness score, as 37.5% were found to be 'more effective' and 62.5% 'less effective'. Interactive technologies (four studies) received an even split of 'more effective' and 'less effective' ratings, both at 50%. In terms of telehealth technologies, only two studies were reviewed, but both received a 'more effective' rating, accounting for a perfect 100% score. Social media technologies, safety monitoring, and medication management technology also had limited studies evaluated (two, two, and one study, respectively). These technologies had an even distribution of 'more effective' and 'less effective' scores, all at 50%. In conclusion, the majority of technologies reviewed showed some level of effectiveness, whether more or less effective. Clearly, further research is needed, particularly for technologies with limited evaluated studies, to provide a more comprehensive understanding of their effectiveness.

### 4.2 Limitations of the review

Although in the review we searched many articles, there is always possibility that some relevant studies were not included because of searching query options (including 60+ older adults, without any specific disease, living at home, having digital solution and evaluation). The search from selected electronic databases (WOS, IEEE Xplore and SCOPUS) was limited to 2010 to January 2024. Although this time interval was selected according to the improvements in health technologies for older adults, it can be possible that some relevant studies may be published before or after that period or in other databases. We included only journal articles and conference publications in our review, and gray literature, unpublished reports, or other reports were not included. A potential limitation of this study is the subjective nature of efficacy assessment. This subjectivity leaves room for debate and re-evaluation of the results and evaluations. While we have utilized a published method for efficacy classification, further research is needed to develop more nuanced and comprehensive methods to gain a more in-depth understanding of the studies. Other significant limitation was reviewing the studies in English language for the results. Thus, publications published in other languages were also not included which may result in a language bias and some missing information from other sources.

## 5. CONCLUSION

Aging in place presents a range of challenges for both caregivers and seniors. These challenges encompass the need for costly home modifications, healthcare expenditures, and the emotional and physical toll on family members or professional caregivers.

Despite the potential of technology to facilitate independent living for community-dwelling older adults, adoption and usage remain limited among this demographic. This gap stems primarily from the design of technology that fails to adequately address the specific needs and preferences of seniors. The development of technology often overlooks the capabilities and aspirations of the aging population, resulting in difficult-to-navigate or unsuitable technologies for community-dwelling older adults.

This paper underscores the imperative of evaluating technological solutions to determine their efficacy in addressing the needs of community-dwelling older adults. It emphasizes the significance of assessing the impact of technology on various facets of their lives, including social engagement, independence, mental well-being, physical health, and safety. Specifically, the paper stresses the role of technology in supporting instrumental activities of daily living (IADLs), which are essential for community-dwelling older adults to maintain independent living. It highlights the importance of identifying technologies that effectively assist in these activities to enhance the quality of life and promote independence among this population.

In conclusion, the review identified a strong emphasis on technology solutions for health and communication IADL categories among the community dwelling older adults. While there is a focus on usability and acceptance, the open-source availability of these solutions remains limited. Future research in this field should consider a broader range of IADL categories and address the challenges associated with technology adoption by the community dwelling older adults.
